# High production of IL-12 by human dendritic cells stimulated with combinations of pattern-recognition receptor agonists

**DOI:** 10.1038/s41541-024-00869-1

**Published:** 2024-05-03

**Authors:** Brian C. Gilmour, Alexandre Corthay, Inger Øynebråten

**Affiliations:** 1 Tumor Immunology Lab, Department of Pathology, Rikshospitalet, Oslo University Hospital, Oslo, Norway; 2 Hybrid Technology Hub – Centre of Excellence, Institute of Basic Medical Sciences, University of Oslo, Oslo, Norway; 3 Institute of Clinical Medicine, University of Oslo, Oslo, Norway

**Keywords:** Cell vaccines, Toll-like receptors, RIG-I-like receptors

## Abstract

The cytokine IL-12p70 is crucial for T helper 1 (Th1) polarization and the generation of type 1 immunity required to fight cancer and pathogens. Therefore, strategies to optimize the production of IL-12p70 by human dendritic cells (DCs) may significantly improve the efficacy of vaccines and immunotherapies. However, the rules governing the production of IL-12p70 remain obscure. Here, we stimulated pattern recognition receptors (PRRs) representing five families of PRRs, to evaluate their ability to elicit high production of IL-12p70 by monocyte-derived DCs. We used ten well-characterized agonists and stimulated DCs in vitro with either single agonists or 27 different combinations. We found that poly(I:C), which engages the RNA-sensing PRRs TLR3 and MDA5, and LPS which stimulates TLR4, were the only agonists that could elicit notable IL-12p70 production when used as single ligands. We identified six different combinations of PRR agonists, all containing either the TLR3/MDA5 agonist poly(I:C) or the TLR7/8 agonist R848, that could synergize to elicit high production of IL-12p70 by human DCs. Five of the six combinations also triggered high production of the antiviral and antitumor cytokine IFNβ. Overall, the tested PRR ligands could be divided into three groups depending on whether they triggered production of both IL-12p70 and IFNβ, only one of the two, or neither. Thus, combinations of PRR agonists were found to increase the production of IL-12p70 by human DCs in a synergistic manner, and we identified six PRR agonist combinations that may represent strong adjuvant candidates, in particular for therapeutic cancer vaccines.

## Introduction

According to the 3-signal model, a naïve T cell is activated upon recognition of cognate antigen wherein antigen-presenting cells (APCs) provide signal 1 and 2 by presenting the peptide-MHC complex and co-stimulatory molecules, respectively, to the T cell^1–4^. Signal 3 is mediated by cytokines that stimulate receptors on the T cell^5–8^. Pattern recognition receptors (PRRs) can contribute to all three signals since APCs stimulated with PRR agonists may up-regulate antigen presentation, and increase the expression of co-stimulatory molecules and signal 3 cytokines^9–12^. Thus, there is a great interest in PRRs as targets of vaccine adjuvants^13–15^.

A successful immune response against intracellular pathogens and cancer is typically associated with a strong T helper 1 (Th1) and CD8^+^ T cell response, i.e. type 1 immunity^16–19^. The cytokine IL-12p70 is crucial for the polarization of naïve CD4^+^ T cells into Th1 cells^7,20,21^, and strategies that can optimize the production of IL-12p70 by dendritic cells (DCs) may therefore significantly improve the efficacy of vaccines and immunotherapies for cancer. However, with the exception of toll-like receptor 3 (TLR3) and TLR4, the ability of different PRRs to trigger IL-12p70 production remains poorly characterized^13,14^.

IL-12p70 is a heterodimer of p35 and p40, which are disulfide-linked and recognized by the IL-12 receptor^22,23^. IL-12p70 synthesis is controlled by several transcription factors of which interferon regulatory factors (IRFs) and NF-κB are the most studied^24–28^. The p35 unit is considered the limiting factor in IL-12p70 production, but how the p35 gene is activated and thereby how IL-12p70 synthesis is regulated, remain obscure^22,29,30^. IL-12p40 can be present as a monomer, homodimer, or a heterodimer where the p35 chain may be substituted with p19 to form IL-23^31^. However, only IL-12p70, i.e. the heterodimer of p35 and p40, is able to polarize naïve CD4^+^ T cells into Th1 cells. Therefore, quantitative data on the p40 unit/IL-12p40, which is presented in numerous studies, is not a reliable measure of IL-12p70. Only specific measurement of the heterodimer of p35 and p40 is representative of the Th1-polarizing cytokine IL-12p70.

PRRs can be divided into several families: i) TLRs; ii) C-type lectin receptors (CLRs); iii) nucleotide-binding oligomerization domain (NOD)-like receptors (NLRs); and iv) retinoic acid-inducible gene (RIG)-I-like receptors (RLRs). In addition, PRRs include cyclic GMP-AMP synthase (cGAS) and the absent in melanoma 2 (AIM2)-like receptors (ALRs) such as IFI16. The TLRs, which constitute the best characterized family of PRRs, recognize a variety of ligands including lipopolysaccharide (LPS), lipoprotein, double stranded RNA (dsRNA) and single stranded RNA (ssRNA), as well as unmethylated CpG-containing double stranded DNA (dsDNA)^32,33^. CLRs sense virus, bacteria, and fungi typically by recognition of the carbohydrate mannose, but CLRs also recognize self-ligands such as F-actin. Depending on the particular CLR-ligand partners, the interaction may lead to cellular immunosuppression or an inflammatory response^34^. NLRs, RLRs, ALRs, and cGAS are all present in the cytosol, but recognize different ligands: NLRs sense intracellular infection and stress, and the first NLRs that were described, NOD1 and NOD2, bind structures from bacterial peptidoglycans^35–37^. RLRs recognize dsRNA and are important in defence against viral infections^38,39^. Finally, cGAS and the ALR IFI16 sense dsDNA^40–42^.

Until now, PRR agonists present in licensed vaccines against human infectious diseases typically target TLRs. For example, *Salmonella Minnesota*-derived Monophosphoryl lipid A that stimulates TLR4 is used in vaccines against papilloma virus and hepatitis B virus^13,43^. The TLR9 agonist oligodeoxynucleotide CpG 1018 is included in the protein subunit-based vaccines against hepatitis B (Heplisav-B) and SARS-CoV-2, and recently, a TLR7/8 agonist (Alhydroxiquim-II) became part of a vaccine against SARS-CoV-2 (COVAXIN)^44^. In cancer immunotherapy, combined stimulation of TLR3 and TLR7/8 has been used in clinical studies to activate monocyte-derived DCs (moDCs)^45,46^. Finally, TLR3 agonists have been tested in numerous preclinical experiments, and some have been transferred to clinical studies^47^. In contrast, the adjuvant potential of other PRR family members is much less characterized.

In this study, we used established PRR agonists to engage receptors covering the different families of PRRs. The ligands were added to cultures of moDCs, and the signal 3 cytokine IL-12p70, the antiviral and antitumor cytokine IFNβ, and the immunosuppressive cytokine IL-10 were quantified in the cell culture media. DC activation was also assessed by measurement of cell surface expression of CCR7, HLA-DR, and the co-stimulatory molecules CD80 and CD86. In total, we used ten ligands to stimulate 11 different PRRs. When DCs were treated with single agonists, only poly(I:C), a synthetic analog of dsRNA that binds to TLR3 and MDA5; and LPS, a ligand of TLR4, induced notable IL-12p70 production. Poly(I:C) or R848 (Resiquimod), a ligand of TLR7/8, in combination with ligands of the cell surface receptors dectin-1 and TLR2:1, respectively, synergized and induced high levels of IL-12p70. The tested PRR ligands and their combinations could be divided into three groups depending on whether they triggered production of both IL-12p70 and IFNβ, only one of the two or neither. We identified six different combinations of PRR agonists that trigger high IL-12p70 production by human DCs and may thereby represent promising vaccine adjuvant candidates.

## Results

### The TLR3/MDA5 agonist poly(I:C) or TLR4 agonist LPS-EK induce IL-12p70 production by human monocyte-derived DCs

To investigate the effect of different PRR agonists on the production of IL-12p70, IFNβ, and IL-10 by human DCs, we isolated CD14^+^ monocytes from healthy blood donors and generated moDCs by culturing the cells in vitro for 6 days with GM-CSF and IL-4 according to an established protocol^45,48^. Flow cytometry analysis showed that CD14 expression was down-regulated during differentiation from monocyte to moDC, which had acquired a typical CD14^lo^, CD11c^hi^, and HLA-DR^hi^ DC phenotype (Supplementary Figs. 1 and 2). With the aim to activate PRRs with different cellular locations and belonging to the different PRR families, we selected ten established PRR agonists for this study (Table 1; additional information provided in Supplementary Table 1). The moDCs were stimulated with the PRR agonists for 24 h in vitro, and the cytokines IL-12p70, IFNβ, and IL-10 released into the cell culture medium were quantified by ELISA.

**Table 1:** Pattern recognition receptors (PRRs) and the respective agonists selected for this study

PRRs	Family^a^	Adaptor	Localization	Microbial ligand	Ligand origin	Test ligand^b^
Dectin-1	CLR	Ras, Syk	Surface	β-glucans	Fungi/bacteria/viruses	Zymosan-D
TLR2:1^c^	TLR	MyD88	Surface	Triacylated lipoprotein^f^	Bacteria	Pam3CSK4
TLR2:1/6^d^	TLR	MyD88	Surface	Diacylated lipoprotein^f^	Bacteria	LTA
TLR3	TLR	TRIF	Endosome	dsRNA	Viruses/Bacteria	poly(I:C)
TLR4	TLR	MyD88, TRIF	Surface, endosome	Lipopolysaccharide	Bacteria/fungi	LPS-EK
TLR7/8	TLR	MyD88	Endosome	ssRNA	Bacteria/viruses	R848
TLR9	TLR	MyD88	Endosome	dsDNA	Bacteria/viruses	CpG
RIG-I	RLR	MAVS	Cytosol	dsRNA	Viruses	3p-hpRNA^g^
MDA5/TLR3^e^	RLR	MAVS	Cytosol	dsRNA	Viruses	poly(I:C)^g^
cGAS		STING	Cytosol	dsDNA	Bacteria/Viruses	poly(dA:dT)^g,h^
IFI16	ALR		Cytosol	dsDNA	Bacteria/viruses	poly(dA:dT)^g,h^
NOD2	NLR	RIP2	Cytosol	Muramyl dipeptide	Bacteria	*N*-GMDP

^a^PRR family: *CLR* C-type lectin receptor, *TLR* toll-like receptor, *RLR* retinoic acid inducible gene I (RIG-I)-like receptor, *ALR* absent in melanoma 2 (AIM2)-like receptor, *NLR* nucleotide-binding oligomerization domain (NOD)-like receptor.

^b^Zymozan-D, zymosan depleted, i.e. purified by hot alkali to remove compounds with TLR-activating properties^70–72^; LTA, lipoteichoic acid; LPS-EK, ultrapure lipopolysaccharide (LPS) from the *E. coli* strain K12; *N*-GMDP, N-glycolyl muramyl dipeptide.

^c^Heterodimer of TLR1 and TLR2^73^.

^d^Heterodimers of TLR2 and TLR1, and TLR2 and TLR6^74,75^.

^e^Transfected poly(I:C) may engage both MDA5 and TLR3.

^f^Molecules from viruses, fungi, and helminths can also be ligands of TLR2 containing heterodimers^76^.

^g^The ligand was transfected into the cytosol by LyoVec^TM^, a lipid-based transfection reagent.

^h^Poly(dA:dT) may be recognized by several cytosolic DNA sensors.

**Fig. 1:**
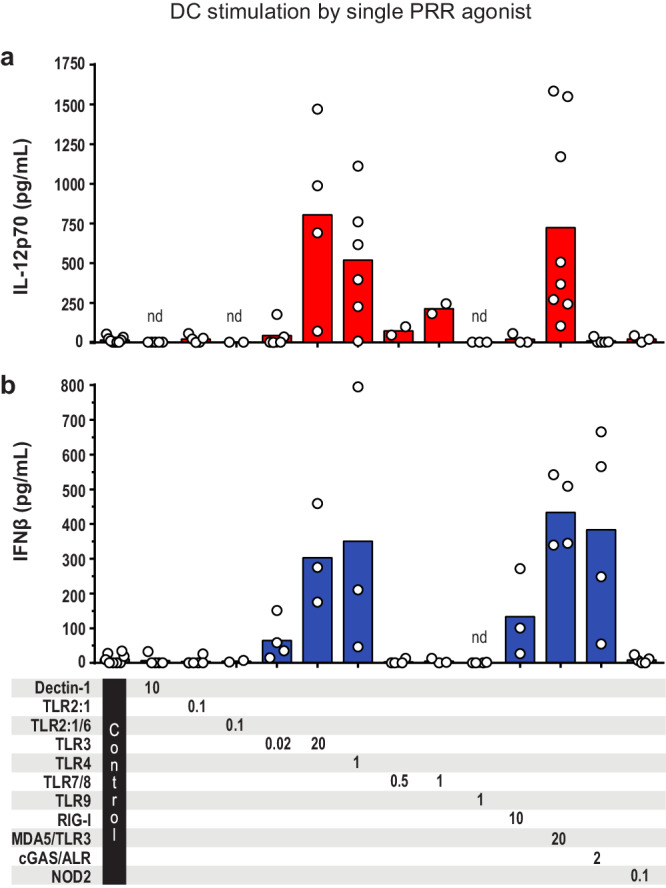
IL-12p70 and IFNβ released from moDCs stimulated with single ligands engaging the indicated PRRs. Cultures of moDCs were stimulated for 24 h with single PRR ligands before **a** IL-12p70, and **b** IFNβ were measured in the cell culture supernatants. Unstimulated moDCs were used as control. Each circle shows the cytokine concentration from one experiment, and the bars present the mean (*n* = 2–9). The numbers below the bars indicate the ligand concentrations in μg/mL. TLR toll-like receptor, RIG-I retinoic acid-inducible gene I, MDA5 melanoma differentiation-associated protein 5, cGAS cyclic GMP-AMP synthase, ALR absent in melanoma 2 (AIM2)-like receptor, NOD2 nucleotide-binding oligomerization domain 2. nd not detectable.

In the first experimental setting, moDCs were stimulated with ten single PRR ligands, and unstimulated moDCs were used as control (Fig. 1). Strikingly, the TLR3 and MDA5 agonist poly(I:C) and the TLR4 agonist LPS-EK were the only ligands able to induce notable and consistent IL-12p70 production by moDCs (Fig. 1a, Supplementary Table 2). In addition, the TLR7/8 agonist R848 (Resiquimod) was found to induce low levels of IL-12p70 production (Fig. 1a). Furthermore, four single PRR agonists were able to induce secretion of IFNβ by moDCs: the TLR3/MDA5 agonist poly(I:C), the TLR4 agonist LPS-EK, the RIG-1 agonist 3p-hpRNA, and the cGAS agonist poly(dA:dT) (Fig. 1b, Supplementary Table 3). In contrast to what we observed for IL-12p70 in some experiments, the TLR7/8 agonist R848 did not elicit IFNβ production (Fig. 1b). Taken together, the data show that only some PRRs are able to trigger the secretion of IL-12p70 and IFNβ by human moDCs. Moreover, there was only a partial overlap between the PRRs that induce notable production of IL-12p70 (TLR3, TLR4, MDA5) and IFNβ (TLR3, TLR4, MDA5, RIG-1, cGAS), suggesting that there are different rules for production of IL-12p70 and IFNβ.

### Six different combinations of PRR agonists, all containing poly(I:C) or R848, elicit high levels of IL-12p70

**Fig. 2:**
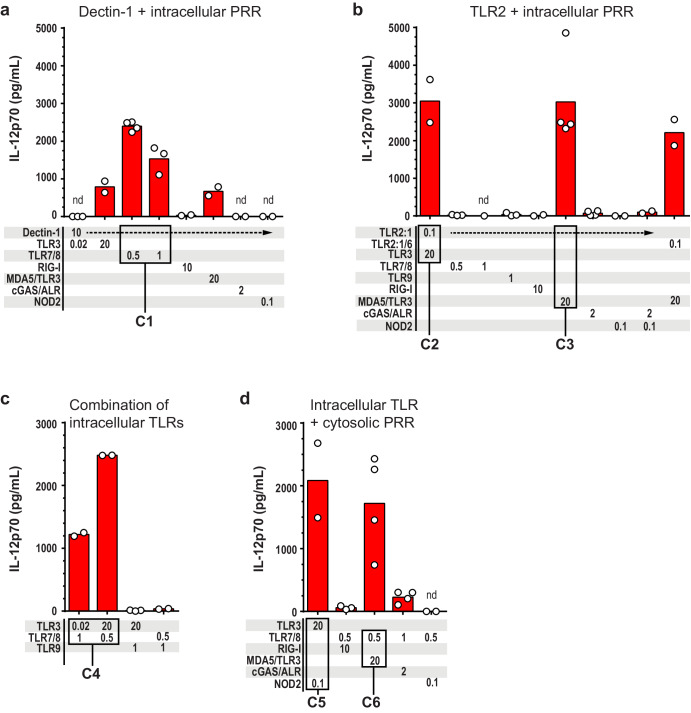
The TLR3 and MDA5 agonist poly(I:C) can be combined with ligands of cell surface PRRs as well as intracellular PRRs to elicit high IL-12p70 production. IL-12p70 was quantified in the cell culture media of moDCs stimulated for 24 h with ligands of the indicated PRRs. Various combinations of PRRs were stimulated: The cell surface PRRs **a** dectin-1 (*n* = 2–4) or **b** TLR2 (*n* = 1–4) in combinations with intracellular PRRs; **c** combinations of intracellular TLRs (*n* = 2–3); and **d** intracellular TLR + cytosolic PRR (*n* = 2–4). The ligand concentrations in μg/mL are given below the bars. Each circle presents the concentration from one experiment, and bars represent the mean. C1–C6 highlight the PRR combinations which resulted in high IL-12p70 levels. nd not detectable.

We reasoned that, in a natural setting, pathogens are likely recognized by several PRRs, and that they may first engage a cell-surface PRR before entering cells and coming in contact with endosomal and/or cytosolic PRRs^49^. We therefore stimulated the cell surface PRRs dectin-1 or TLR2 in combination with an intracellular PRR (Fig. 2a, b). In other experiments, combinations of intracellular PRRs were stimulated (Fig. 2c, d). When dectin-1 was stimulated by use of zymosan-D, only the combinations containing ligands of TLR3, MDA5, or TLR7/8 resulted in moderate-to-high amounts of IL-12p70 (Fig. 2a). Based on the levels of IL-12p70 that were generated, we considered dectin-1 + TLR7/8 to be a potent combination and named it C1 (Fig. 2a). Whereas dectin-1 and TLR7/8 separately induced non-detectable and at the most, low levels of IL-12p70, respectively, the combination C1 resulted in >2000 pg/mL IL-12p70, revealing a synergistic effect upon stimulation of the two PRRs (Figs. 1a and 2a).

Pam_3_CSK_4_, which is an agonist of the cell surface PRR TLR2:1, induced high production of IL-12p70 only when combined with poly(I:C), either non-transfected (TLR3) or transfected (MDA5/TLR3) (Fig. 2b). These combinations are designated C2 and C3. Stimulation of TLR2:1 in combination with the remaining PRRs, including TLR7/8, resulted in barely detectable or non-detectable levels of IL-12p70 (Fig. 2b). Thus, whereas dectin-1 could be stimulated together with TLR3, MDA5, or TLR7/8 to trigger production of IL-12p70, TLR2:1-stimulation resulted in high levels of IL-12p70 only when it was combined with poly(I:C) (TLR3, MDA5).

Combined stimulation of intracellular PRRs by use of poly(I:C) (TLR3) and R848 (TLR7/8) induced high production of IL-12p70 (combination C4; Fig. 2c). TLR3 stimulation could also be combined with NOD2 (C5), and TLR7/8 stimulation could be combined with MDA5/TLR3 (C6) to induce high IL-12p70 production (Fig. 2d). Taken together, poly(I:C), the ligand of TLR3 and MDA5, could be combined with any of the tested ligands of intracellular PRRs (with the exception of TLR9) and elicit production of IL-12p70.

Finally, we tested combinations which included the TLR4 ligand LPS-EK (Supplementary Fig. 3a, b). TLR4 differs from the other PRRs because it can signal both from the cell surface and from an intracellular site, the endosome, and thereby activate two distinct signaling pathways^50,51^. All combinations which contained LPS-EK, elicited IL-12p70 production (Supplementary Fig. 3a, b).

### Combinations of two agonists synergize to induce high production of IL-12p70

**Fig. 3:**
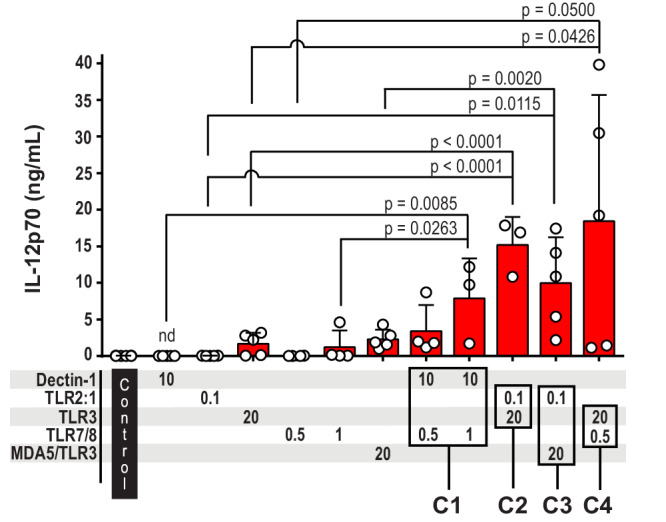
Combined stimulation with two PRR ligands synergistically increase IL-12p70 production. Cultures of moDCs were stimulated for 24 h with the previously identified ligand combinations (C1, C2, C3, and C4) along with the respective single ligands. Unstimulated moDCs were set up as control. IL-12p70 was quantified in the cell culture supernatants, and each circle shows the concentration from one experiment. The ligand concentrations in μg/mL are indicated below the bars. Bars present the mean with SD (*n* = 3–6). *P* values < 0.05 were considered statistically significant, and were calculated by ANOVA using Dunnettˈs test to correct for multiple comparisons. nd, not detectable.

We performed side-by-side comparisons of the IL-12p70 levels induced by single PRR agonist versus PRR agonist combinations. We tested four of the combinations which in the first experiments demonstrated high IL-12p70 production: C1 (dectin-1 + TLR7/8), C2 (TLR2:1 + TLR3), C3 (TLR2:1 + MDA5/TLR3), and C4 (TLR3 + TLR7/8) (Figs. 2a–c and 3). All the tested combinations (C1–C4) showed a synergistic effect on the level of IL-12p70 (Fig. 3). Synergy was most pronounced for dectin-1 + TLR7/8 (C1), which in comparison to the separate PRRs increased the IL-12p70 level more than 100-fold (Table 2). Moreover, combinations containing the TLR4 ligand LPS-EK induced IL-12p70 levels that were comparable to those triggered by C1–C4 (Supplementary Fig. 3). In summary, selected PRRs can be combined to synergistically increase the IL-12p70 production by human DCs.

**Table 2:** Fold increase of IL-12p70 concentrations in response to the PRR combinations C1–C4 as compared to their respective single ligands

Stimulated PRRs	Fold increase, IL-12p70^a^
Combination C1, Dectin-1 + TLR7/8	
Dectin-1	≥170
TLR7/8-0.5^b^	≥125
Combination C1, Dectin-1 + TLR7/8	
Dectin-1	≥390
TLR7/8-1^c^	6.4
Combination C2, TLR2:1 + TLR3	
TLR2:1	≥400
TLR3	9
Combination C3, TLR2:1 + MDA5/TLR3	
TLR2:1	≥260
MDA5/TLR3	4.3
Combination C4, TLR3 + TLR7/8^d^	
TLR3	11
TLR7/8	≥680

^a^Fold increase of IL-12p70 levels was calculated from the results presented in Fig. 3.

Samples with non-detectable level of IL-12p70, were given the value of the detection limit to calculate the fold increase. For these samples, the fold increase could potentially be higher, and fold increase has been set as “≥“.

^b^The TLR7/8 agonist R848 was used in concentration 0.5 μg/mL.

^c^The TLR7/8 agonist R848 was used in concentration 1 μg/mL.

^d^Poly(I:C) (TLR3) and R848 (TLR7/8) were used in concentration 20 μg/mL and 0.5 μg/mL, respectively.

### The combinations that include an agonist of TLR3, TLR4, RLR, or cGAS, induce IFNβ production

**Fig. 4:**
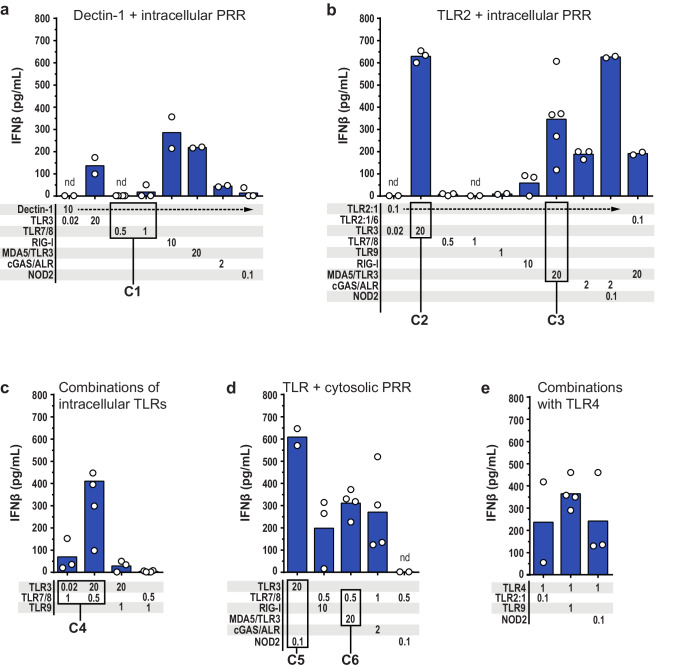
Several combinations of PRR ligands induce substantial levels of IFNβ. The indicated PRRs were stimulated for 24 h before IFNβ was quantified in the cell culture media of moDCs. Various combinations of PRRs were stimulated: The cell surface PRRs **a** dectin-1 (*n* = 2–4) or **b** TLR2 (*n* = 2–5) in combination with intracellular PRRs; **c** combinations of intracellular TLRs (*n* = 3–4); **d** intracellular TLR + cytosolic PRR (*n* = 2–4); and **e** combinations including TLR4 (*n* = 2–4). The concentrations of the PRR ligands are indicated in μg/mL below the bars. Each circle represents the IFNβ concentration from one experiment, and bars show the mean. The combinations C1–C6 induced high production of IL-12p70. nd not detectable.

Next, we quantified the IFNβ levels in response to the same conditions that were tested for the ability to induce IL-12p70 production (Fig. 4). Strikingly, the combination C1 (dectin-1 + TLR7/8), which triggered high production of IL-12p70 (Figs. 2a and 3), resulted in minor to non-detectable levels of IFNβ (Fig. 4a). In contrast, stimulation of dectin-1 could be combined with stimulation of several other intracellular PRRs (TLR3, RIG-I, MDA5, or cGAS) to elicit IFNβ production (Fig. 4a). Moreover, both C2 (TLR2:1 + TLR3) and C3 (TLR2:1 + MDA5/TLR3), as well as stimulation of TLR2:1 in combination with RIG-1 or cGAS induced IFNβ production (Fig. 4b). Thus, IFNβ was produced when stimulation of the cell surface PRRs dectin-1 or TLR2:1 was combined with stimulation of an intracellular PRR that alone was able to elicit IFNβ production (Figs. 1b and 4a, b).

IFNβ was also produced in response to several of the combinations which stimulated intracellular PRRs, such as C4 (TLR3 + TLR7/8), C5 (TLR3 + NOD2), and C6 (TLR7/8 + MDA5/TLR3) (Fig. 4c, d). Moreover, all tested combinations containing the TLR4 agonist LPS-EK, induced IFNβ production (Fig. 4e). Taken together, the data presented in Fig. 4 show that the combinations which included an intracellular PRR recognizing DNA or RNA, or TLR4 sensing LPS, elicited IFNβ production. Among the combinations C1-6, which induced high levels of IL-12p70, all except C1 induced substantial production of IFNβ.

### Stimulation with a single PRR ligand is sufficient to induce high production of IFNβ

**Fig. 5:**
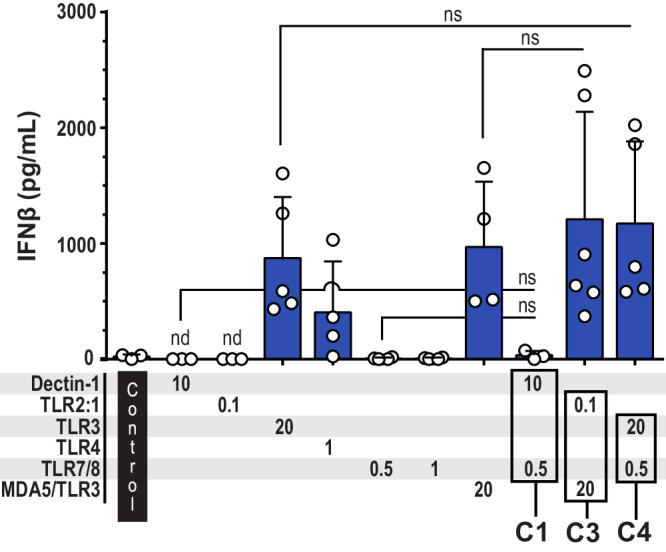
Stimulation with two PRR ligands elicits IFNβ levels similar to that of the respective single ligands. Cultures of moDCs were stimulated for 24 h with ligand combinations C1, C3, and C4 along with the respective single ligands, before IFNβ was quantified in the cell culture supernatants. The used PRR ligand concentrations are indicated in μg/mL below the respective bars. Each circle shows the IFNβ concentration of one experiment, and bars represent the mean with SD (*n* = 3–6). *P* values were calculated by ANOVA using Dunnettˈs test to correct for multiple comparisons, and *p* values < 0.05 were considered statistically significant. Control, unstimulated moDCs. nd not detectable, ns not significant.

Finally, we tested whether the combinations dectin-1 + TLR7/8 (C1), TLR2:1 + MDA5/TLR3 (C3), or TLR3 + TLR7/8 (C4) could act in synergy and increase the level of IFNβ (Fig. 5). In accordance with what we had observed already (Fig. 4a), stimulation of dectin-1 + TLR7/8 (C1) resulted in IFNβ levels similar to that measured in media from unstimulated moDCs (Control), i.e. the levels were minor (Fig. 5). The response to LPS-EK (TLR4) varied between samples of moDCs, but the IFNβ level was not significantly different from that induced by poly(I:C) (TLR3; MDA5/TLR3). Stimulation of TLR2:1 + MDA5/TLR3 (C3) or TLR3 + TLR7/8 (C4) generated IFNβ levels in the same range as stimulation of MDA5 or TLR3 with poly(I:C) (Fig. 5). Thus, the combinations C1, C3, and C4 showed no synergistic effect on the level of IFNβ, and stimulation with a single PRR ligand is sufficient to induce high production of IFNβ.

### PRR stimulation did not raise the level of IL-10

**Fig. 6:**
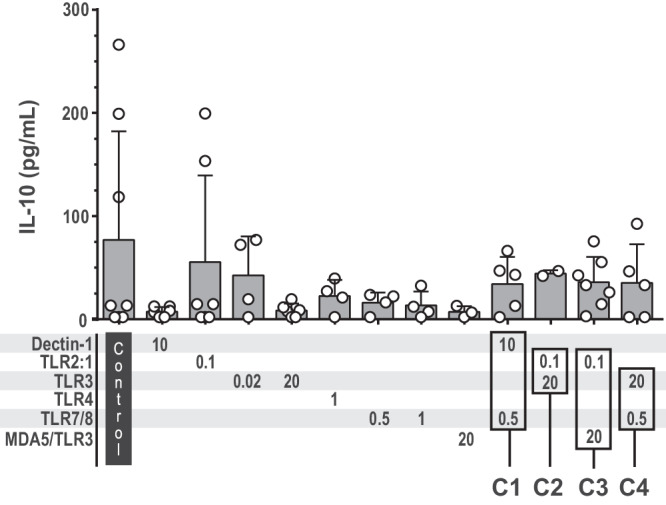
Stimulation of PRRs did not increase the amount of released IL-10 from moDCs. The moDCs were left unstimulated (control) or stimulated for 24 h before IL-10 was quantified in the cell culture supernatants. Each circle presents the concentration from one experiment, and bars represent the mean with SD (*n* = 2–8). ANOVA did not reveal significant differences between the groups. The combinations C1, C2, C3, and C4 were tested because they elicited high levels of IL-12p70. The numbers below the bars indicate the concentrations (μg/mL) of the PRR ligands.

Because IL-10 is an anti-inflammatory cytokine that can inhibit T cell activation and memory development, we examined whether a selection of PRR agonists, including the combinations C1–C4, had an influence on the level of IL-10 produced by moDCs (Fig. 6). In two experiments, relatively high levels of IL-10 were measured in cell culture media from unstimulated moDCs and from moDCs stimulated with the TLR2:1 ligand. However, in an average from 4–7 experiments, the tested PRR ligands, either alone or in combinations, resulted in IL-10 levels similar to that measured in unstimulated moDC cultures (Fig. 6).

### The combination of TLR2:1 and TLR3/MDA5 agonists significantly increased the surface expression of CCR7, CD80, CD86, and HLA-DR

**Fig. 7:**
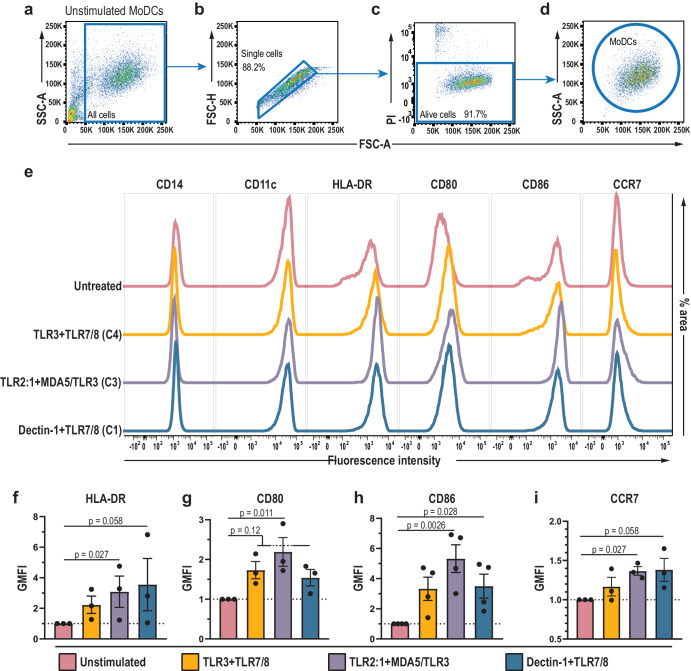
Stimulation of TLR2:1 + MDA5/TLR3 increase the surface expression of HLA-DR, CD80, CD86, and CCR7 on moDCs. Cultures of moDCs were stimulated with ligands of Dectin-1 + TLR7/8 (C1), TLR2:1 + MDA5/TLR3 (C3), or TLR3-0.02 μg/mL + TLR7/8-1 μg/mL (C4) for 24 h before analysis by flow cytometry. Unstimulated moDCs were used as control. **a**–**d** Before analysis of the surface molecules, single, live moDCs were identified as indicated: **a** Debris was excluded by size (FSC-A), and **b** single cells were selected and doublets excluded in an FSC-A vs. FSC-H plot, before **c**, **d** the live cells, defined as propidium iodide (PI)-negative were selected for analysis of cell surface molecules. **e** Surface expression by moDCs of CD14, CD11c, HLA-DR, CD80, CD86, and CCR7 in a representative sample. CD14 and CD11c showed similar expression levels at all tested conditions whereas PRR stimulation resulted in a significant increase or tendency towards increase in surface expression of the indicated molecules. Geometric mean fluorescence intensity (GMFI) was calculated for **f** HLA-DR, **g** CD80, **h** CD86, and **i** CCR7 and compared to unstimulated moDCs whose GMFI was set to 1. The bars indicate mean values with SD, and each dot represents the result from one experiment (*n* = 3–4). *P* values were calculated by use of an uncorrected Dunn’s Test, and *p* < 0.05 was considered statistically significant.

The tested ligands and respective PRRs could be divided into three groups depending on whether they triggered production of both IL-12p70 and IFNβ, only one of the two or neither (Table 3; for table with colors, see Supplementary Table 4). Among these, stimulation of TLR2:1 + TLR3 (C2), TLR2:1 + MDA5/TLR3 (C3), TLR3 + TLR7/8 (C4), or TLR3 + NOD2 (C5) resulted in particularly high IL-12p70 and IFNβ production (Table 3). The combination dectin-1 + TLR7/8 (C1) stood out as it induced high levels of IL-12p70 but minor-to-non-detectable levels of IFNβ (Table 3). For further analysis, we selected C1, C3, and C4 because they are expected to trigger different signaling pathways and they showed differences in IFNβ expression (Figs. 4a–c and 5). Moreover, C4 (TLR3-0.02 μg/mL + TLR7/8-1 μg/mL) was included because this combination is used to activate moDCs for therapeutic cancer vaccination^46,52^. We examined the ability of C1, C3, and C4 to up-regulate the surface expression of four DC activation markers, i.e. the chemokine receptor CCR7, the co-stimulatory molecules CD80 and CD86, and the MHC class II molecule HLA-DR (Fig. 7). The cell surface expression of these molecules was analyzed by flow cytometry using a gated single cell, live population of moDCs (Fig. 7a–d). C3 (TLR2:1 + MDA5/TLR3) induced a significant increase in the cell surface expression of all four activation markers (Fig. 7e–i), and C1 (dectin-1 + TLR7/8) up-regulated the expression of CD86 (Fig. 7e, h). The remaining test conditions resulted in a tendency to increased surface expression of the activation markers (Fig. 7e–i). Taken together, the combinations C1, C3, and C4 which induced high IL-12p70 production, elicited a trend or significant increase in cell surface expression of the lymph node homing receptor CCR7 and of the molecules CD80, CD86, and HLA-DR which are pivotal in the contribution to signal 1 and signal 2 during T cell activation.

**Table 3:** Data summary for the production of IL-12p70 and IFNβ by DCs stimulated with PRR agonists

Stimulated PRRs^a^	Ligands	IL-12p70^b^	IFNβ^b^
C1: Dectin-1 + TLR7/8	Zymosan-D + R848	+++++	−/+
C2: TLR2:1 + TLR3	Pam3CSK4 + poly(I:C)	+++++	+++
C3: TLR2:1 + MDA5/TLR3^c^	Pam3CSK4 + poly(I:C)^d^	+++++	+++
C4: TLR3 + TLR7/8	poly(I:C) + R848	+++++	+++
C5: TLR3 + NOD2	poly(I:C) + *N*-GMDP	+++++	+++
C6: TLR7/8 + MDA5/TLR3^c^	R848 + poly(I:C)^d^	+++++	++
TLR3 + TLR7/8	poly(I:C) [0.02]^e^ + R848	++++	+
Dectin-1	Zymosan-D	−/+	−/+
TLR2:1	Pam3CSK4	−/+	−/+
TLR2:1/6	LTA	−/+	−/+
TLR3	poly(I:C) [0.02]^e^	+	+
TLR3	poly(I:C)	++++	+++
TLR4	LPS-EK	+++	+++
TLR7/8	R848	+	−/+
TLR9	CpG	−/+	−/+
RIG-I	3p-hpRNA^d^	−/+	+
MDA5/TLR3^c^	poly(I:C)^d^	++++	+++
cGAS/ALR^f^	poly(dA:dT)^d^	−/+	+++
NOD2	*N*-GMDP	−/+	−/+
Unstimulated	No ligand	−/+	−/+

^a^C1–C6 indicate the identified ligand combinations that induce high production of IL-12p70 by moDCs.

^b^Graded concentrations of IL-12p70 and IFNβ in the media from stimulated moDCs: −/+ indicates non-detectable to 20 pg/mL cytokine; +, 21–100 pg/mL; ++, 101–350 pg/mL; +++, 351–800 pg/mL; ++++, 801–1500 pg/mL; and +++++, >1500 pg/mL.

^c^Poly(I:C) was transfected by lipid-based transfection and may engage both MDA5 and TLR3.

^d^Lipid-based transfection was used to transfer the ligand into the cytosol.

^e^Poly(I:C) concentration: 0.02 μg/mL. When not indicated, poly(I:C) was used in concentration 20 μg/mL.

^f^Several sensors can recognize dsDNA: cGAS, and ALRs (e.g. IFI16 and DAI).

## Discussion

In this study, we stimulated human moDCs from different donors with ligands of receptors from all five families of PRRs, with the aim to identify the different PRRsʼ ability to induce production of the Th1-polarizing cytokine IL-12p70, and the antiviral and antitumor cytokine IFNβ. When using single agonists, many of the intracellular PRRs elicited IFNβ production (TLR3, TLR4, RIG-I, MDA5, and cGAS; but not TLR7/8, TLR9, or NOD2), whereas only poly(I:C), a ligand of both TLR3 and MDA5, and the TLR4 ligand LPS, induced substantial production of IL-12p70. The tested cell surface PRRs dectin-1, TLR2:1, and TLR2:1/6 did neither elicit IL-12p70 nor IFNβ production. Notably, all ten tested PRR ligands demonstrated biological activity, and those that were unable to induce cytokine production when used as single ligands, showed biological activity in combinations with other PRR agonists. The PRR ligands that we tested could be divided into three groups depending on whether they induced production of both IL-12p70 and IFNβ, only one of the two or neither. Among 27 tested combinations of different PRR ligands, six combinations, which either contained poly(I:C) or R848 (Resiquimod) that engage RNA-sensing PRRs, elicited high IL-12p70 production. The six identified PRR ligand combinations may represent strong adjuvant candidates for vaccines.

A key question that we wanted to address was whether one signal is sufficient to trigger notable IL-12p70 production by human DCs, or whether two signals are required. Among ten tested single agonists, only poly(I:C) elicited IL-12p70 production in all experiments; LPS-EK increased the IL-12p70 level in all except one experiment, and R848 (1 μg/mL) induced some IL-12p70 production in four out of six experiments. The high molecular weight poly(I:C) (1.5–8 kb long) that we used is an established agonist of TLR3 and MDA5, whereas 5’-triphosphate hairpin RNA (89 bp) engages RIG-1^53–55^. To stimulate MDA5 and RIG-I, which are localized in the cytosol, the ligands were transfected into the moDCs. We were surprised by the finding that only poly(I:C), and not the short hairpin RNA, elicited IL-12p70 production since activated MDA5 and RIG-I have been reported to trigger similar signaling pathways, i.e. to interact with mitochondrial antiviral-signaling protein (MAVS), and activate IRF3, IRF7, and NF-κB^38,56^. Although, it cannot be excluded that the MDA5 and RIG-I signaling pathways may be similar but not identical, which could potentially explain the discrepancy in triggering IL-12p70 production, our results made us wonder whether transfected poly(I:C) not only stimulated MDA5 but also TLR3. Some transfected poly(I:C) may potentially have triggered endosomal TLR3; and vice versa, when aiming to stimulate TLR3 in the absence of transfection reagent, some poly(I:C) may possibly enter the cytosol and activate MDA5. Thus, poly(I:C) (with or without transfection) may engage both TLR3 and MDA5, which potentially could synergize to elicit production of IL-12p70. Moreover, R848, which induced low levels of IL-12p70 in some experiments, is also an agonist of two receptors (TLR7 and TLR8), which are reported to activate distinct signaling pathways^57^. Finally, although LPS is a ligand of a single receptor, TLR4, it is well established that TLR4 signals via the adaptor molecules MyD88 and TRIF, which activate two different signaling pathways (NF-κB and IRF, respectively)^27,51^. Thus, although IL-12p70 production could be triggered by the single ligands poly(I:C), R848, or LPS, it is possible that two signals, i.e. stimulation of two distinct signaling pathways may be required for IL-12p70 production.

Our data show that combinations of two PRR ligands can increase the level of IL-12p70 in a synergistic manner. The combination zymosan-D + R848 (C1, dectin-1 + TLR7/8) induced a pronounced effect and increased the IL-12p70 level >100-fold compared to the TLR7/8 ligand alone. The combinations C2 (TLR2:1 + TLR3) and C4 (TLR3 + TLR7/8) increased the IL-12p70 level about 10-fold compared to poly(I:C) alone. A 4.3-fold increase of the IL-12p70 level was observed for C3 (TLR2:1 + MDA5/TLR3) in comparison to MDA5/TLR3 alone. Furthermore, although C5 (TLR3 + NOD2) and C6 (TLR7/8 + MDA5/TLR3) were not compared side by side to their respective single ligands in the same experiment, both combinations triggered much higher IL-12p70 levels as compared to the single ligands, strongly suggesting a synergistic effect. Taken together, our data indicate that stimulation with two different PAMPs is required to trigger high levels of IL-12p70 production by moDCs. However, any combination of PAMPs did not result in enhanced IL-12p70 production. Among the 27 different combinations tested, only the combination zymosan-D + R848 (C1, dectin-1 + TLR7/8) and the combinations which contained poly(I:C) (except for poly(I:C)+CpG) or LPS elicited high IL-12p70 production. Notably, all combinations which induced high IL-12p70 production either contained an agonist of RNA-sensing PRRs (TLR3, MDA5, or TLR7/8) or TLR4-binding LPS. Because a high level of IL-12p70 is expected to be beneficial for the induction of type I immunity that is critical to fight pathogens and cancer, our data suggest that the identified combinations of two PRR agonists should be more potent as vaccine adjuvant as compared to single agonists.

In accordance with our findings, it has been previously reported that some PRR agonists such as poly(I:C) + R848 and LPS + R848 can synergize to increase IL-12p70 production by moDCs^26,58,59^. Interestingly, a combination of selected PRR agonists (poly(I:C) + R837) was also reported to synergistically increase IL-6 production by BDCA1^+^ DCs^60^. A model was proposed in which type I IFN secreted in response to poly(I:C)-mediated TLR3 activation, acts in an autocrine manner on DCs and induces de novo expression of TLR7. Then, R837-mediated activation of the newly expressed TLR7 synergizes with the original TLR3 activation and amplifies cytokine production^60^. Thus, PRR synergy appears not to be restricted to IL-12p70. However, the mechanism of synergy could differ for monomeric cytokines (such as IL-6) and IL-12p70, because IL-12p70 consists of two subunits whose expression is regulated by different transcription factors^22,24,25,27^. Moreover, the synergistic effect on the IL-6 level appeared to be less than 1.5-fold based on the presented mean values^60^. In contrast, synergy induced by the PRR ligand combinations in our study increased the amount of IL-12p70 by at least 4-fold (C3, TLR2:1 + MDA5/TLR3) and >100-fold for C1–C4 as compared to TLR2:1 or dectin-1.

Here, we examined the effect of PRR agonists on human moDCs. It should be noted that in vitro activated moDCs are commonly used as DC vaccines to treat patients with cancer because classical DCs (cDCs) are rare in blood and thereby difficult to isolate in sufficient numbers^46,61–63^. However, when PRR agonists are delivered directly in vivo as a vaccine adjuvant instead, various DC subsets, such as cDC type I (cDC1), cDC2, and DC3, may potentially be exposed to the PRR ligands. Interestingly, it has been shown that a combination of two PRR agonists (poly(I:C) + TLR8 ligand) synergistically increased IL-12p70 production ex vivo in a pool of enriched blood DCs^64^. Moreover, R848 + LPS was reported to synergistically increase IL-12p70 production by CD1c^+^ DCs (cDC2)^65^, and a combination of PRR agonists (poly(I:C) + R848 + LPS) was shown to induce notable production of IL-12p70 by both DC2 and DC3^66,67^. Thus, although further studies are needed to clarify the rules for IL-12p70 production across DC subsets, it is tempting to speculate that the need for two signals to trigger high production of IL-12p70 also applies to cDC1, cDC2, and DC3.

Further studies are needed to investigate the downstream effects on T cell responses of DC stimulation with the hereby identified ligand combinations C1–C6. Notably, in two previous studies, although IL-12p70 levels were not investigated, it was shown that combinations of PRR agonists could enhance and sustain the Th1-polarizing capacity of moDCs (LPS + R848), and increase the T cell-derived IFNγ production (poly(I:C) + TLR8 ligand TL8-506)^26,64^. Moreover, IL-12p70 in moDC-T cell co-cultures was shown to be important for high expression of the cytolytic molecule granzyme B in CD8^+^ T cells^68^. Finally, Okada et al., reported that the levels of IL-12p70 produced by moDCs used for vaccination in glioblastoma patients in a phase I/II study positively correlated with time to progression^69^. Thus there are several studies pointing towards a beneficial effect of PRR synergy and IL-12p70 on T cell responses.

Our findings may be important for the design of adjuvants in vaccines and cancer immunotherapy. The six identified PRR agonist combinations (C1–C6) all induced high levels of IL-12p70 by human DCs, and, except for C1 (dectin-1 + TLR7/8), elicited IFNβ production. PRR synergy to ensure consistent, high IL-12p70 production across patients may be a viable strategy to promote protective Th1 responses and thereby type I immunity that is critical to successfully eliminate pathogens and cancer.

## Methods

### Positive selection of human monocytes

Monocytes were isolated from buffy coats obtained from the blood bank at Oslo University Hospital, Oslo, Norway, and approved for use by the Norwegian Regional Committee for Medical and Health Research Ethics, REK no. 2019/113. The buffy coats were diluted 1:1 with sterile phosphate buffered saline without Mg^2+^ and Ca^2+^ (PBS^−/−^) containing 2% fetal bovine serum (FBS) (Biowest, #S181BH), before gently being added on top of Lymphoprep^™^ (Progen, #1114545) in 50 mL tubes in volumes recommended by the provider. The tubes were centrifuged at 800 × *g* for 20 min at room temperature (RT) with the brake disabled. Peripheral blood mononuclear cells (PBMCs) located at the interface of the plasma and Lymphoprep layers were collected and washed twice in PBS^−/−^ with 2% FBS by centrifugation (400 × *g*, 7 min at RT) to remove remnants of Lymphoprep. The pelleted PBMCs were re-suspended in PBS^−/−^ with 2% FBS and passed through a 30 μm filter (Miltenyi, #130-041-407) to remove cell clumps and debris. Next, the monocytes were positively selected from the PBMCs by magnetic-activated cell sorting (MACS) technology using CD14^+^ MicroBeads (Miltenyi, #130-050-201) according to the manufacturer’s instructions: PBMCs, per 10^7^ cells, were re-suspended in 80 μL cold MACS buffer (PBS^−/−^ with 10% FBS and 2 mM EDTA) and mixed with 20 μL MicroBeads, to a maximum volume of 2 mL in the presence of higher cell numbers. The mixture of PBMCs and MicroBeads was vortexed before incubation for 15 min at 4 °C. Next, after washing of the cells, maximum 1 ×10^8^ labeled cells were applied onto an LS column (Miltenyi, #130-090-976) placed in a MACS magnet separator (Miltenyi, #130-042-109). Unlabeled cells were washed out before the magnetically labeled cells were harvested in 5 mL PBS^−/−^ with 2% FBS by use of a plunger. Staining with an APC/Cy7-conjugated anti-human CD14 antibody (clone HCD14) followed by flow cytometry, showed that >95% of the positively selected cells were monocytes (*n* = 3). The positively selected monocytes were either frozen in FBS containing 10% DMSO (PanReac AppliChem, #67-68-5) or immediately differentiated by GM-CSF and IL-4 as described below.

### Differentiation protocol for human monocyte-derived dendritic cells

On day 0, 2–2.5 × 10^6^ frozen or freshly isolated monocytes were seeded in 10 cm non-tissue culture treated dishes (VWR, #734-2796) in 10 mL RPMI 1640 (Biowest, #L0500) with 10% FBS (Biowest, #S181BH), 1% Penicillin-Streptomycin (Biowest, #L0022), 100 ng/mL GM-CSF (PeproTech, #300-03) and 20 ng/mL IL-4 (PeproTech, #200-04). The cells were cultivated at 37 °C and 5% CO_2_. On day 3, medium was replenished by adding 10 mL fresh medium containing 100 ng/mL GM-CSF and 20 ng/mL IL-4. At day 5, the cells were harvested and seeded out for use in experiments. Non-adhesive cells were collected directly from the medium. Loosely adherent cells were harvested by applying 5 mL cold PBS^−/−^ to the dish for 5–10 min at RT before harvesting the cells by pipetting the PBS^−/−^ carefully up and down on the plate 3–5 times.

### Stimulation with pattern-recognition receptor ligands

Harvested day 5 moDCs were plated in 96-well plates (Corning, #3595) (120 000 cells in 100 μL/well) for quantification of released cytokines, and in non-tissue culture treated 6-well plates (Corning, #3736) (500 000 cells in 1.4 mL/well) for flow cytometry analysis of cell surface molecules. RPMI 1640 with 10% FBS, 1% Penicillin-Streptomycin, 100 ng/mL GM-CSF, and 20 ng/mL IL-4 was used as medium. After cultivation for 24 h at 37 °C and 5% CO_2_, PRR ligands (see Table 1, and Supplementary Table 1 for detailed information about the ligands) were added to the moDC cultures (in 6× concentration to the 96-well plates to a total volume of 120 μL). To target cytosolic PRRs, LyoVec^™^ reagent (Invivogen, #lyec-12) was used to transfect the ligands 3p-hpRNA (RIG-I), poly(I:C) (MDA5/TLR3), and p(dA:dT) (cGAS/ALR) into the cytosol according to manufacturer’s protocol. Briefly, the desired ligand was diluted in LyoVec^™^ and incubated for at least 15 min to a maximum of 1 h. Next, the mixture was combined 1:1 with RPMI 1640 containing 10% FBS, 1% Penicillin-Streptomycin and, alternatively another PRR ligand. MoDCs were then incubated with the mixture for 24 h.

For cytokine quantification, the culture medium was harvested from 96-well plates. Cells and debris were removed from the culture medium by centrifugation two times at 4 °C, first at 400 × *g* for 7 min, and then at 1000 × *g* for 15 min. The supernatants were then frozen and stored at −80 °C until analysis.

### ELISA

IFNβ, IL-10, and IL-12p70 were quantified by use of ELISA kits (Supplementary Table 5), carried out in half-area, flat-bottom 96-well plates (Corning, #3690). Excluding PBS^−/−^, all necessary diluents and buffers were from the DuoSet^®^ Ancillary Reagent Kit 2 (R&D Systems, #DY008). The assays were performed at RT unless otherwise noted, and all steps were followed by washing using 200 μL/well of wash buffer (R&D Systems) three times. The desired capture antibody (Supplementary Table 5) was diluted to the concentration recommended by the provider, and 50 μL of the antibody was applied per well before incubation overnight. Next, the wells were blocked using 150 μL/well of reagent diluent (PBS^−/−^ with 1% bovine serum albumin (BSA)) for at least 1 h. Afterwards, 50 μL/well of samples, or standard in 2-fold dilution series were added in duplicates. The plate was incubated for 2 h and washed, before incubation for another 2 h with 50 μL/well of biotinylated detection antibody. Streptavidin-HRP diluted 1:40 (50 μL/well) was incubated for 20 min. After washing, 50 μL/well of a 1:1 solution of H_2_O_2_ (R&D Systems, part #895000) and tetramethylbenzidine (R&D Systems, part #895001) was added for approx. 20 min before the reaction was stopped by addition of 25 μL/well of 2 N H_2_SO_4_. Absorbance was measured at 450 nm and 540 nm (reference wavelength) using an Epoch^™^ Microplate Spectrophotometer, with the 540 nm readings subtracted from the 450 before analysis. Standard curves were made by fitting a four parameter logistic (4PL) curve model to the data.

### Flow cytometry of surface molecules

Harvested cells were kept on ice for approx. 15 min, before 200,000 cells re-suspended in flow buffer (PBS^−/−^ with 10% FBS) were portioned out per staining condition. Fc-receptors were blocked by adding 2.5 μL Human TruStain^™^ FcX (BioLegend, #101320) per 50 μL of 200 000 cells, and after incubation for 30 min on ice, the cells were pelleted by centrifugation (400 × *g*, 7 min at 4 °C). Next, the moDCs were re-suspended in 50 μL flow buffer containing 2.5 μL of fluorophore-conjugated antibody (see Supplementary Table 6 for information about clone, dilution, catalog no., and provider). Irrelevantly targeted antibodies, but otherwise matched to the specific antibodies with regard to fluorophore, concentration, isotype, and species were used as isotype controls (see Supplementary Table 7 for information about clone, dilution, catalog no., and provider). Following incubation for 20 min in the dark on ice, unbound antibody was removed by washing in flow buffer. Finally, the moDCs were re-suspended in 200 μL flow buffer. Immediately before analysis on a BD LSRFortessa^™^ flow cytometer, 2.5 μL propidium iodide (PI) (BioLegend, #421301) was added enabling the elimination of dead cells. eBioscience^™^ UltraComp eBeads (Invitrogen, #01-2222-42) were used to adjust the settings for compensation. Isotype controls and unstained cells were used to set the threshold for positive staining. Data was analyzed using FlowJo V10 software (Becton Dickinson and Company), and geometric mean fluorescent intensity (GMFI) values were exported for further analysis.

### Reporting summary

Further information on research design is available in the Nature Research Reporting Summary linked to this article.

### Supplementary information

Supplementary Material

REPORTING SUMMARY

## Data Availability

The data that support the findings of this study are available from the corresponding author upon reasonable request.
