# Supplementary material for: High production of IL-12 by human dendritic cells stimulated with combinations of pattern-recognition receptor agonists
Source: NPJ Vaccines. 2024 May 3;9:83. doi: 10.1038/s41541-024-00869-1 (PMC11068792; doi:10.1038/s41541-024-00869-1)
Supplement: Supplementary file 1 — Supplementary Material [file 41541_2024_869_MOESM1_ESM.pdf]

*Supplementary Material*

# High production of IL-12 by human dendritic cells stimulated with combinations of pattern-recognition receptor agonists

Brian C Gilmour, Alexandre Corthay, and Inger Øynebråten

## Table of contents

***Supplementary Table 1.*** Ligands used to stimulate pattern recognition receptors (PRRs), and ligand abbreviations ........ 2

***Supplementary Table 2.*** Increased concentrations of the agonists of dectin-1, TLR2:1, or TLR2:6 did not trigger IL-12p70 production ........ 3

***Supplementary Table 3.*** Increased concentrations of the ligands of dectin-1, TLR2:1, or TLR2:6 did not trigger IFNβ production ........ 3

***Supplementary Table 4.*** Table 3 in colors: Data summary for the production of IL-12p70 and IFNβ by DCs stimulated with pattern-recognition receptor agonists ........ 4

***Supplementary Table 5.*** ELISA kits used for quantification of cytokines ........ 5

***Supplementary Table 6.*** Primary antibodies used for analysis of expression of cell surface molecules on monocytes and monocyte-derived DCs ........ 5

***Supplementary Table 7.*** Isotype- and fluorophore-matched, irrelevantly targeted antibodies used as negative controls ........ 5

***Supplementary Figure 1.*** Gating and flow cytometry analysis of monocytes and monocyte-derived DCs ........ 6

***Supplementary Figure 2.*** The blood donors gave rise to monocyte-derived DCs with low CD14 expression ........ 7

***Supplementary Figure 3.*** Levels of IL-12p70 induced by combinations containing the TLR4 ligand LPS-EK ........ 8

**Supplementary Table 1. Ligands used to stimulate pattern recognition receptors (PRRs), and ligand abbreviations**

| PRR ligand | Abbreviation | Target receptor | Tested conc. (µg/mL) | Catno. | Provider |
|---|---|---|---|---|---|
| 5’-triphosphate hairpin RNA[¤] | 3p-hpRNA | RIG-I | 10 | tlrl-hprna | InvivoGen |
| Lipopolysaccharide from *E. coli* K12 (Ultrapure) | LPS | TLR4 | 1 | tlrl-peklps | InvivoGen |
| Lipoteichoic acid from *Staphylococcus aureus* | LTA | TLR2:1, TLR2:6 | 0.1-100 | tlrl-pslta | InvivoGen |
| *N*-Glycolyl-muramyl dipeptide | GMDP | NOD2 | 0.1 | tlrl-gmdp | InvivoGen |
| ODN2395 | CpG | TLR9 | 1 | tlrl-2395 | InvivoGen |
| Pam3CSK4 | Pam3 | TLR2:1 | 0.1-10 | tlrl-pms | InvivoGen |
| Poly(dA:dT) naked[¤] | p(dA:dT) | cGAS/ALR | 2 | tlrl-patn | InvivoGen |
| Poly(I:C) HMW* | poly(I:C) | TLR3 | 0.02; 20 | tlrl-pic | InvivoGen |
| Poly(I:C) HMW[¤]* | poly(I:C) | MDA5/TLR3 | 20 | tlrl-pic | InvivoGen |
| Resiquimod | R848 | TLR7/8 | 0.5; 1 | tlrl-r848 | InvivoGen |
| Zymosan (depleted) | Zym-D | Dectin-1 | 1-20 | tlrl-zyd | InvivoGen |

[¤] 5’-triphosphate hairpin RNA, poly(dA:dT) naked, and poly(I:C) HMW were mixed with LyoVec™ to transfect the ligands into the cytosol.

* HMW, high molecular weight

**Supplementary Table 2. Increased concentrations of the agonists of dectin-1, TLR2:1, or TLR2:6 did not trigger IL-12p70 production**

| Stimulated PRR | Ligand | Ligand concentration (µg/mL) | Absorbance | | IL-12p70 (pg/mL) ¤ |
|---|---|---|---|---|---|
| **Unstimulated** | None | 0 | 0.081 | 0.078 | ND |
| **Dectin-1** | Zym-D | 1 | 0.083 | 0.081 | ND |
| | | 10 | 0.083 | 0.081 | ND |
| | | 20 | 0.080 | 0.083 | ND |
| **TLR2:1** | Pam3CSK4 | 0.5 | 0.088 | 0.082 | ND |
| | | 1 | 0.080 | 0.080 | ND |
| | | 10 | 0.083 | 0.078 | ND |
| **TLR2:1; TLR2:6** | LTA | 0.5 | 0.083 | 0.078 | ND |
| | | 1 | 0.079 | 0.079 | ND |
| | | 10 | 0.084 | 0.080 | ND |
| | | 100 | 0.082 | 0.081 | ND |
| **MDA5/TLR3** | poly(I:C)* | 20 | 1.353 | 1.455 | 1470.5 |

ND, not detectable

¤ Amounts of IL-12p70 (pg/mL) in supernatants from monocyte-derived DCs stimulated with the indicated ligand concentrations for 24 h.

* Poly(I:C) was transfected into the cytosol by LyoVec™, a lipid-based transfection reagent. The ligand was included as positive control.

**Supplementary Table 3. Increased concentrations of the ligands of dectin-1, TLR2:1, or TLR2:6 did not trigger IFNβ production**

| Stimulated PRR | Ligand | Ligand concentration (µg/mL) | IFNβ (pg/mL) ¤ | |
|---|---|---|---|---|
| **Unstimulated** | None | 0 | 7.1 | 7.5 |
| **Dectin-1** | Zym-D | 1 | 7.1 | 7.4 |
| | | 10 | 6.9 | 7.0 |
| | | 20 | 6.9 | 6.7 |
| **TLR2:1** | Pam3CSK4 | 0.5 | 7.1 | 7.6 |
| | | 1 | 6.9 | 7.1 |
| | | 10 | 7.0 | 7.2 |
| **TLR2:1, TLR2:6** | LTA | 0.5 | 7.1 | 7.0 |
| | | 1 | 7.1 | 7.1 |
| | | 10 | 7.1 | 7.8 |
| | | 100 | 7.1 | 7.2 |
| **MDA5/TLR3** | poly(I:C) * | 20 | 261.9 | 289.2 |

¤ Amounts of IFNβ (pg/mL) in supernatants from monocyte-derived DCs stimulated with the indicated ligand concentrations for 24 h.

* Poly(I:C) was transfected into the cytosol by LyoVec™, a lipid-based transfection reagent. The ligand was included as positive control.

**Supplementary Table 4. Table 3 in colors:**
**Data summary for the production of IL-12p70 and IFNβ**
**by DCs stimulated with pattern-recognition receptor agonists**

| Stimulated PRRs [#] | Ligands | IL-12p70 [¤] | IFNβ [¤] |
|---|---|---|---|
| **C1:** Dectin-1 + TLR7/8 | Zymosan-D + R848 | +++++ | -/+ |
| **C2:** TLR2:1 + TLR3 | Pam3CSK4 + poly(I:C) | +++++ | +++ |
| **C3:** TLR2:1 + MDA5/TLR3[§] | Pam3CSK4 + poly(I:C)[¶] | +++++ | +++ |
| **C4:** TLR3 + TLR7/8 | poly(I:C) + R848 | +++++ | +++ |
| **C5:** TLR3 + NOD2 | poly(I:C) + *N*-GMDP | +++++ | +++ |
| **C6:** TLR7/8 + MDA5/TLR3[§] | R848 + poly(I:C)[¶] | +++++ | ++ |
| TLR3 + TLR7/8 | poly(I:C) [0.02]ǂ + R848 | ++++ | + |
| Dectin-1 | Zymosan-D | -/+ | -/+ |
| TLR2:1 | Pam3CSK4 | -/+ | -/+ |
| TLR2:1/6 | LTA | -/+ | -/+ |
| TLR3 | poly(I:C) [0.02]ǂ | + | + |
| TLR3 | poly(I:C) | ++++ | +++ |
| TLR4 | LPS-EK | +++ | +++ |
| TLR7/8 | R848 | + | -/+ |
| TLR9 | CpG | -/+ | -/+ |
| RIG-I | 3p-hpRNA[¶] | -/+ | + |
| MDA5/TLR3[§] | poly(I:C)[¶] | ++++ | +++ |
| cGAS/ALR[‡] | poly(dA:dT)[¶] | -/+ | +++ |
| NOD2 | *N*-GMDP | -/+ | -/+ |
| Unstimulated | No ligand | -/+ | -/+ |

[#] C1-C6 indicate the identified ligand combinations that induce high production of IL-12p70 by moDCs.

[¤] Graded concentrations of IL-12p70 and IFNβ in the media from stimulated moDCs: -/+ indicates non-detectable to 20 pg/mL cytokine; +, 21-100 pg/mL; ++, 101-350 pg/mL; +++, 351-800 pg/mL; ++++, 801-1500 pg/mL; and +++++, > 1500 pg/mL.

[§] Poly(I:C) was transfected by lipid-based transfection and may engage both MDA5 and TLR3.

[¶] Lipid-based transfection was used to transfer the ligand into the cytosol.

ǂ Poly(I:C) concentration: 0.02 µg/mL. When not indicated, poly(I:C) was used in concentration 20 µg/mL.

[‡] Several sensors can recognize dsDNA: cGAS, and ALRs (*e.g.* IFI16 and DAI).

**Supplementary Table 5. ELISA kits used for quantification of cytokines**

| Item | Provider | Catno. |
|---|---|---|
| DuoSet® Human IL-12 p70 | Bio-Techne | DY1270 |
| DuoSet® Human IFN-β | Bio-Techne | DY814-05 |
| DuoSet® Human IL-10 | Bio-Techne | DY217B |

**Supplementary Table 6. Primary antibodies used for analysis of expression of cell surface molecules on monocytes and monocyte-derived DCs**

| Specificity | Fluorophore | Clone | Isotype | Final conc. µg/mL | Provider | Catno. |
|---|---|---|---|---|---|---|
| **CD14** | APC/Cy7 | HCD14 | Mouse IgG1,κ | 20 | BioLegend | 325620 |
| **CD11c** | Pacific Blue | 3.9 | Mouse IgG1,κ | 20 | BioLegend | 301626 |
| **HLA-DR** | BV605 | L243 | Mouse IgG2a,κ | 5 | BioLegend | 307640 |
| **CD80** | PE/Cy7 | 2D10 | Mouse IgG1,κ | 10 | BioLegend | 305217 |
| **CD86** | APC | IT2.2 | Mouse IgG2b,κ | 10 | BioLegend | 305411 |
| **CCR7** | PE/Cy7 | G043H7 | Mouse IgG2a,κ | 10 | BioLegend | 353225 |

**Supplementary Table 7. Isotype- and fluorophore-matched, irrelevantly targeted antibodies used as negative controls**

| Fluorophore | Isotype | Clone | Sepcificity | Provider | Catno. |
|---|---|---|---|---|---|
| **APC/Cy7** | Mouse IgG1,κ | MOPC-21 | Unknown | BioLegend | 400128 |
| **Pacific Blue** | Mouse IgG1,κ | MOPC-21 | Unknown | BioLegend | 400151 |
| **BV605** | Mouse IgG2a,κ | MOPC-173 | Unknown | BioLegend | 400270 |
| **PE/Cy7** | Mouse IgG1,κ | MOPC-21 | Unknown | BioLegend | 400126 |
| **APC** | Mouse IgG2b,κ | MG2b-57 | Trinitrophenol + KLH | BioLegend | 401210 |
| **PE/Cy7** | Mouse IgG2a,κ | MOPC-173 | Unknown | BioLegend | 400231 |

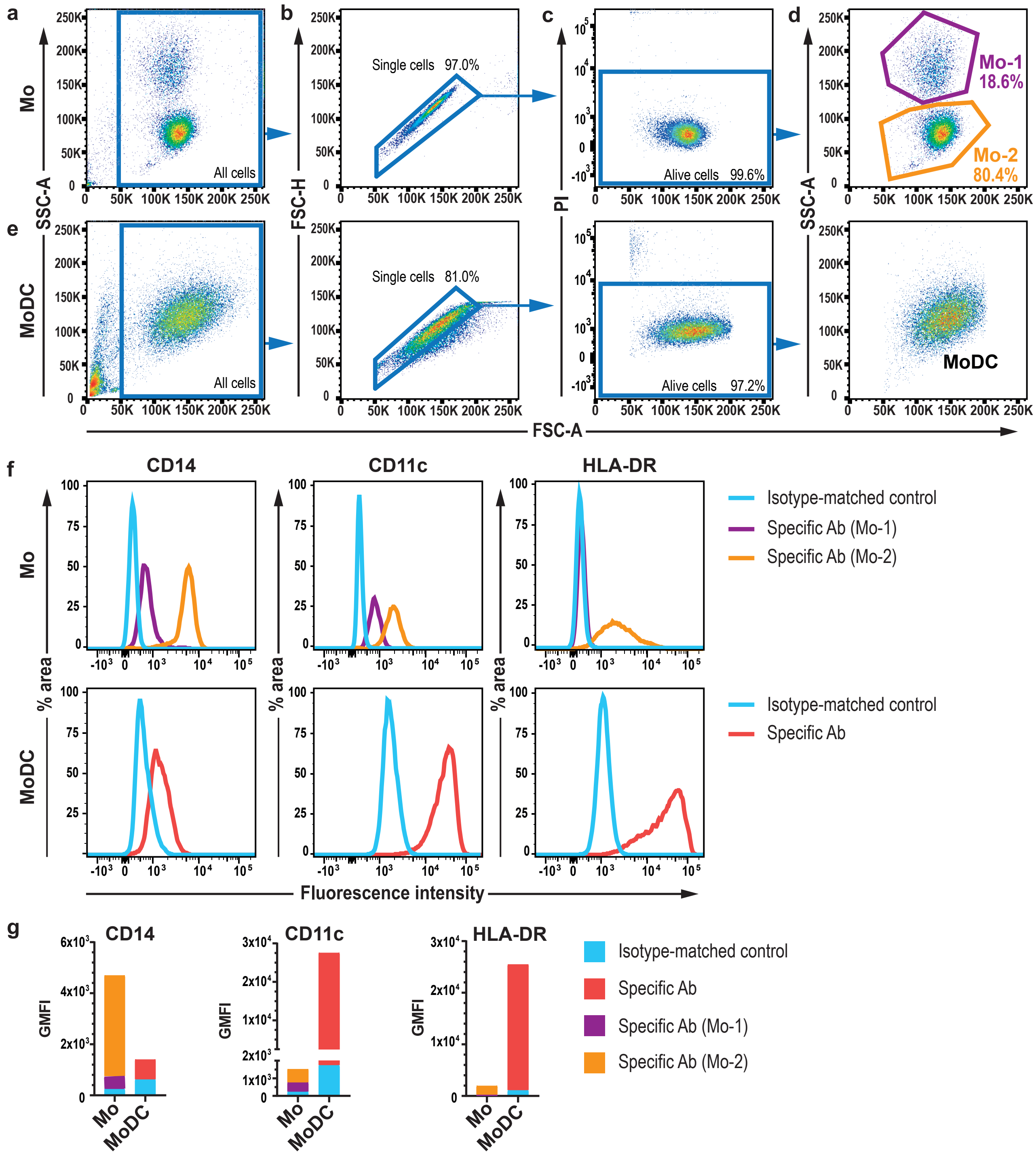


**Supplementary Figure 1. Gating and flow cytometry analysis of monocytes and monocyte-derived DCs.** Positively selected, freshly isolated CD14+ monocytes and monocytes differentiated for 5 days by GM-CSF and IL-4 into monocyte-derived dendritic cells (moDC) were gated and analysed by flow cytometry as indicated: **a** The monocytes (Mo) were first gated based on size and complexity in a forward scatter (FSC-A) and side scatter (SSC-A) plot; **b** Next, a FSC-A and FSC-H plot was used to gate single cells and exclude doublets; **c** Dead cells were removed by their uptake of propidium iodide (PI); **d** Gated live, single cells distributed in two populations, Mo-1 and Mo-2, which constituted 18.6 and 80.4 % respectively, of the gated monocytes. **e** The same gating strategy was used for moDCs, and gated live, single moDCs distributed in one population in the FSC-A and SSC-A plot. **f** Mo-1 showed low CD14 and CD11c expression, and was negative for HLA-DR. Mo-2 on the other hand, was positive for CD14, CD11c, and HLA-DR. The moDCs showed reduced expression of CD14 compared to Mo-2, and increased expression of CD11c and HLA-DR compared to both Mo-1 and Mo-2. **g** The graphs show geometric mean fluorescence intensity (GMFI) for CD14, CD11c, and HLA-DR calculated from the experiment shown in f. The specific antibodies that were used: anti-CD14, clone HCD14; anti-CD11c, clone 3.9; and anti-HLA-DR, clone L243. The antibodies were used alongside isotype- and concentration-matched controls.

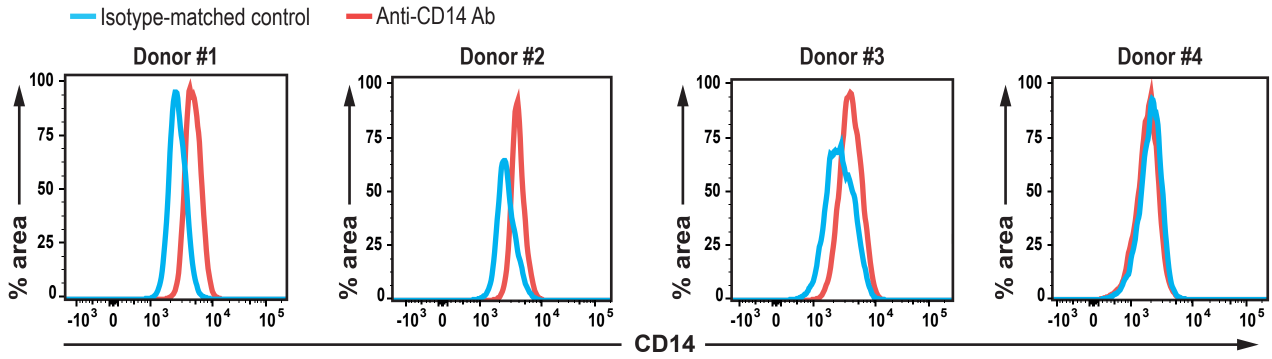


**Supplementary Figure 2. The blood donors gave rise to monocyte-derived DCs with low CD14 expression.** Monocyte-derived dendritic cells (moDCs) were differentiated with GM-CSF (100 ng/mL) and IL-4 (20 ng/mL) for 5 days before flow cytometry analysis of CD14. The moDCs were gated as indicated in Supplementary Figure 1 before evaluation of CD14 expression. The control antibody (blue line, clone MOPC-21) matched the isotype and concentration of the anti-CD14 antibody (red line, clone HCD14). MoDCs generated from different donors (Donor 1-4), showed low to no expression of CD14, suggesting that the monocytes were successfully differentiated into moDCs. The proportion of CD14+ moDCs varied from 13.8-31.8 % with an average value of 22 % across the donors.

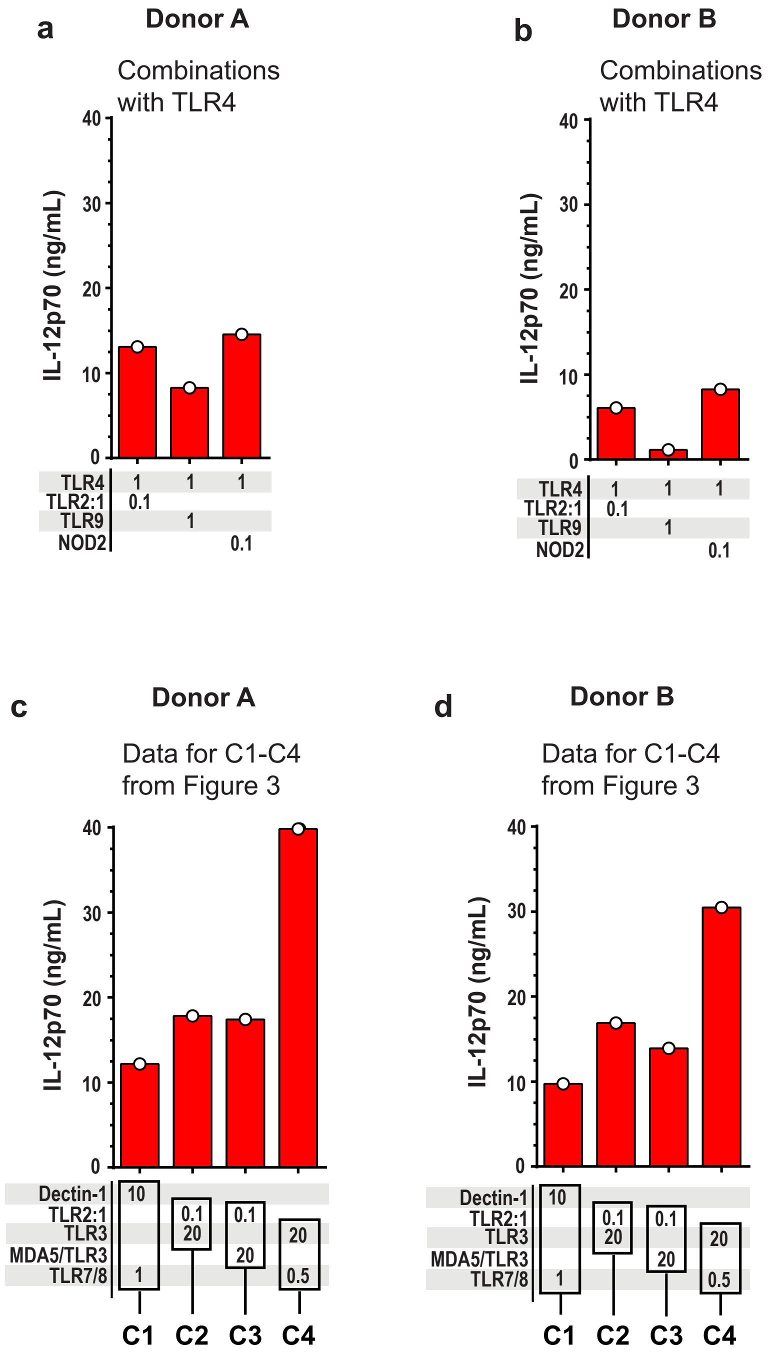


**Supplementary Figure 3. Levels of IL-12p70 induced by combinations containing the TLR4 ligand LPS-EK.** MoDC from two donors were stimulated for 24 h with different combinations of ligands: **a**, **b** LPS-EK (TLR4) in combination with an agonist of TLR2:1, TLR9 or NOD2; and **c**, **d** the combinations C1, C2, C3, and C4. IL-12p70 was quantified in the cell culture supernatants. The concentrations of the PRR ligands are indicated in µg/mL below the bars. Each bar present one experiment from one donor. The data presented in c and d for the combinations C1-C4 are part of Figure 3.
